# Label-free Detection of Influenza Viruses using a Reduced Graphene Oxide-based Electrochemical Immunosensor Integrated with a Microfluidic Platform

**DOI:** 10.1038/srep42771

**Published:** 2017-02-15

**Authors:** Renu Singh, Seongkyeol Hong, Jaesung Jang

**Affiliations:** 1 School of Mechanical and Nuclear Engineering, Ulsan National Institute of Science and Technology (UNIST), Ulsan 44919, Republic of Korea; 2 Department of Biomedical Engineering, UNIST, Ulsan 44919, Republic of Korea

## Abstract

Reduced graphene oxide (RGO) has recently gained considerable attention for use in electrochemical biosensing applications due to its outstanding conducting properties and large surface area. This report presents a novel microfluidic chip integrated with an RGO-based electrochemical immunosensor for label-free detection of an influenza virus, H1N1. Three microelectrodes were fabricated on a glass substrate using the photolithographic technique, and the working electrode was functionalized using RGO and monoclonal antibodies specific to the virus. These chips were integrated with polydimethylsiloxane microchannels. Structural and morphological characterizations were performed using X-ray photoelectron spectroscopy and scanning electron microscopy. Electrochemical studies revealed good selectivity and an enhanced detection limit of 0.5 PFU mL^−1^, where the chronoamperometric current increased linearly with H1N1 virus concentration within the range of 1 to 10^4^ PFU mL^−1^ (R^2^ = 0.99). This microfluidic immunosensor can provide a promising platform for effective detection of biomolecules using minute samples.

Human influenza A H1N1 virus is a serious global health concern^1^, and in February 2010 more them 16,000 cumulative deaths were reported from 213 countries according to the World Health Organization^2^. Influenza virus infection occurs via inhalation of virus-laden particles or direct contact with virus-contaminated surfaces, and is characterized by acute respiratory infection symptoms such as high fever, lethargy, and coughing^3^,^4^. As this virus spreads rapidly via air transmission and infections can be fatal to humans, early and accurate diagnosis is crucial for proper medical treatment and prevention of further infections. The conventional diagnostic techniques for H1N1 virus include plaque assay, enzyme-linked immunosorbent assay, real-time polymerase chain reaction (PCR), reverse transcriptase-PCR, immunoblotting, and immunofluorescence assays^5^. Although these methods are widely used for clinical diagnosis, they need time-consuming sample preparation, expensive reagents and equipment, and trained personnel^5^. Moreover, these techniques are not apt for developing countries due to the expensive processing costs and limited access to pathological laboratories.

To overcome these limitations, several different kinds of biosensors have been developed. Among these, electrochemical biosensors have several advantages: simple instrumentation, fast response time, high sensitivity, simplicity, compatibility with miniaturized and portable systems, and high signal-to-noise ratio^6^,^7^.

Although many studies on electrochemical biosensors have been performed, there have been limited reports on electrochemical detection of the influenza virus H1N1 so far. Pedersen *et al*.^8^ fabricated a DNA aptamer based microelectrode array by utilizing functionalized conductive polymer ((2,3-dihy- drothieno[3,4-b][1,4]-dioxin-2-yl) methanol) (PEDOT-OH:TsO) and it could detect 10 PFU mL^−1^ in the dynamic range of 10–10^6^ PFU mL^−1^ in ~15 min via electrochemical impedance spectroscopy (EIS) technique. Zhang *et al*.^9^ used a glucose sensor by exploiting the enzyme, neuraminidase (NA), and found the limit (10^2^ PFU) and range (10^2^–10^8^ PFU in 1 hr) of detection for this first-generation assay. A boron-doped diamond electrode terminated with a sialic acid-mimic peptide was used to detect H1N1 and H3N2 influenza viruses and the detection range of 20–500 PFU was found^10^.

Graphene has gained considerable research attention in recent years, owing to its extraordinary mechanical, thermal, and electrical properties^11^. Due to these outstanding properties, graphene has been used in fabrication of several devices, such as field-effect transistors^12^, ultrasensitive sensors^13^, and electrochemical resonators^14^. Reduced graphene oxide (RGO), a derivative of graphene, has found many applications in biosensing, drug delivery, and cellular imaging, owing to its excellent biocompatibility, large surface area, ease-of-synthesis, superior water dispersibility, high mobility of charge carriers, high electric conductivity, and high mechanical strength which make it an ideal candidate to integrate with electrochemical devices^15^,^16^. Zhou *et al*.^17^ utilized RGO as an effective biosensing system based on the greatly enhanced electrochemical reactivity of purine and pyrimidine bases, and detected single-nucleotide polymorphism without hybridization, labeling processes, or the use of electrochemical mediators or indicators. They utilized the superior electrocatalytic activity of RGO toward H_2_O_2_ and NADH for detection of glucose and ethanol, exhibiting favorable electron transfer kinetics for the electrocatalysis of neurotransmitter and other biological molecules^17^.

In this study, we report a novel RGO-coated electrochemical immunosensor, integrated with a microfluidic platform for effective detection of an influenza A H1N1 virus in a label-free manner. To the best of our knowledge, microfluidics-based electrochemical detection of a virus using graphene sheets has never been explored before. The effective detection was implemented via N-ethyl-N-(3-dimethylaminopropyl) carbodiimide/N-hydroxysuccinimide (EDC/NHS) coupling for improving immobilization of biomolecules through the direct linkage between carboxyl groups on an RGO surface and amino groups on an antibody (Ab), without an intervening linker or spacer^18^. Furthermore, the large surface area of RGO facilitates many defects and electroactive sites, thereby increasing sensitivity and selectivity. This electrochemical immunosensor was integrated with a microfluidic platform, offering many advantages over macroelectrode-based electrochemical cells^19^,^20^. In fact, influenza viruses can be collected into a quite amount of collection buffer from a large air volume, and a microfluidic sensor platform can make it possible to effectively detect these viruses in the collection buffer^21^,^22^. Further, the present immunosensor detects whole viruses, rather than their nucleic acids, allowing for simple sample preparation. Three microelectrodes were fabricated on a glass substrate using the conventional microfabrication technique, functionalized with biomolecules, and encapsulated with a polydimethylsiloxane (PDMS) microchannel (Fig. 1). Electrochemical detection of H1N1 viruses was then performed at different virus concentrations, from 1 to 10^4^ PFU mL^−1^.

## Results and Discussion

Figure 2(a) shows transmission electron micrographs of GO before (i, ii) and after (iii, iv) conversion into RGO sheets. These images clearly indicate graphene sheets, with some of them overlapped. It is evident that the prepared GO and RGO were transparent, wrinkled, and owing to its high surface tension, flake-like structures with partially folded or scrolled edges emerged to maintain their planarity^23^,^24^. The bright and dark areas show thin and thick layers of RGO, respectively. The high-resolution transmission electron microscopy (TEM) images (iii, iv) show that the prepared RGO samples are few layers (approximately 6–8 layers) thick nanosheets.

Very well-distinct D, G, and 2D bands were observed in the Raman spectra of GO (spectra i) and RGO (spectra ii) (Fig. 2(b)). After reduction of GO, the D = G intensity of the resultant RGO decreased, suggesting that crystalline sp2 domains were formed within RGO flakes^25^. Further analysis of RAMAN spectra is shown in Supplementary Data (section 3).

A wavelength range of 200–800 nm with 2 nm width was used to carry out the UV-visible measurements for GO and RGO (Fig. 2(c)). The UV-VIS spectra of GO exhibited a maximum absorption peak at approximately 225 nm (curve i), due to π-π* transition of aromatic C-C bonds of GO, and the peak was red-shifted towards 270 nm for RGO. This shifting in the absorption peak indicates the reduction of GO (curve ii)^26^.

Atomic force microscopy (AFM) and scanning electron microscopy (SEM) were used to characterize the surface morphology of the RGO sheets before and after virus capture (Fig. 3). As revealed by AFM (Fig. 3(a)), the RGO sheet was smooth with a thickness of ~0.35 nm, corresponding to a multi-layered structure, which agreed with the TEM results. The existence of residual O_2_-containing functional groups around the RGO sheets resulted in the large thickness^27^. These sheets showed lateral size dimensions of 2 to 5 μm. Surface roughness of the RGO/CA/Au surface was 45.4 nm, as seen in Fig. 3(a). Figure 3(b) shows a magnified image of the RGO sheets shown in Fig. 3(a), and exhibited a surface roughness of 6.69 nm. Owing to its high surface tension, wrinkled and flake-like structures with partially folded or scrolled edges (black lines) emerged and were responsible for maintaining the planarity of sheets (Fig. 3(c))^23^,^24^. Few of the sheets were overlapped. Figure 3(d) shows an aggregate of various (~5) influenza viruses (diameter: ~625 nm) captured by antibodies considering the size of a single influenza virus (80–120 nm)^28^.

Figure 4(a) shows the wide-scan spectra measured by X-ray photoelectron spectrometry (XPS) for RGO/CA/Au (i) and Ab/RGO/CA/Au (ii) electrodes. RGO showed the peaks at 285.3 and 532.6 eV for the C1s and O1s, respectively. The characteristic peak for N1s was observed at 440.2 eV (spectrum (ii)).

Table S1 contains the atomic concentrations (%) of C1s, N1s, O1s, and S2p. The atomic concentration of N1s for the Ab/RGO/CA/Au electrode was found to be 6.41%. This result indicates that the RGO/CA/Au surface facilitated the covalent functionalization of antibody via EDC-NHS chemistry.

The Shirley-type baseline and Lorentzian-Doniac-Sunsic curves with a Gaussian profile were used to deconvolute the peaks for the C1s region of RGO/CA/Au and Ab/RGO/CA/Au electrodes (Fig. 4(b,c)). The peaks observed at 284.6, 285.6, 286.6, 287.1, and 288.6 eV correspond to graphitic (C–C), hydroxyl (C–OH), epoxy(C–O), carbonyl (C=O), and carboxylic acid (O=C-O) groups present in RGO, respectively (Fig. 4(b)). In the Ab/RGO/CA/Au electrode (Fig. 4(c)), the binding energy peaks were almost similar to the spectra of the RGO/CA/Au electrode. However, there were decrease in the intensities and slight shifts in the peaks, which can be due to the covalent immobilization of antibody to RGO matrix (Fig. 4(c) and Fig. S3(a)), and an extra prominent wide peak at 287.9 eV can be responsible for the formation of amide bond (Co-NH) between RGO matrix and antibody (Fig. 4(b))^29^. Figure S3 shows the XPS analysis for the N1s spectra of RGO/CA/Au and Ab/RGO/CA/Au electrodes. The peak aroused at 398.9 eV due to the nitrogen atoms proves the immobilization of antibody on to the RGO/CA/Au surface (Fig. S3 (b)). The deconvoluted peaks for the N1s region of the Ab/RGO/CA/Au electrode with Gaussian fitting at 401.5 eV exactly confirms the covalent immobilization of antibody (Fig. S3 (c))^30^.

The electrochemical behaviors of CA/Au (curve i), RGO/CA/Au (curve ii), Ab/RGO/CA/Au (curve iii), and BSA/Ab/RGO/CA/Au (curve iv) electrodes were investigated using cyclic voltammetry (CV) in 10 mM PBS (pH 7.4) containing 2.5 mM [Fe(CN)_6_]^3−/4−^ and 100 mM NaCl. The scan rate and flow rate were 50 mV/s and 130 μL min^−1^, respectively (Fig. 5(a)). The cystamine (CA) layer deposited onto the Au electrode showed a very small current due to the insulating layer of CA. The anodic peak current increased up to 8.5 μA for the RGO/CA/Au electrodes, which is attributed to the superior electrical conductivity, large surface area, and unique electron transport property of RGO which could accelerate the electron transfer rate and enhance the redox conversion at the electrode/electrolyte interface^31^. It has already been found that edge-plane of the RGO exhibits a heterogenous electron transfer rate (ke) of the order of ∼0.01 cm/s, and the basal plane is effectively inert, with kb (basel plane) lower than 10^−9^ cm/s due to the presence of abundant oxygen containing groups (carboxyl groups)^32^, which make it more hydrophilic and highly dispersible in water for better immobilization of antibody^17^,^33^. The anodic peak current for Ab/RGO/CA/Au electrodes was found to decrease (2.82 μA) because of the redox active sites deeply entrapped within the antibodies, obstructing the electron transfer. Fascinatingly, blocking of the redox active sites available on the electrode resulted in the considerable decrease in the current observed for BSA/Ab/RGO/CA/Au electrodes. Figures 5b and 5c show cyclic voltammograms of the RGO/CA/Au and Ab/RGO/CA/Au electrodes, respectively, for scan rates of 10–100 mV s^−1^. As we increased the scan rate the anodic and cathodic peak potentials were noticed to be shifted towards positive and negative axis, respectively. However, the fast electron exchange was acquired by the linear increase in the redox peak currents with square root of the scan rate, which was due to the controlled reaction by semi-infinite linear diffusion^34^. The anodic and cathodic peak currents for the RGO/CA/Au electrodes were given by Eqs (1) and (2), respectively:


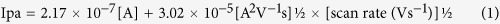



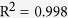



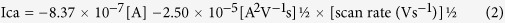



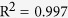


As for the Ab/RGO/CA/Au electrodes, the anodic and cathodic peak currents were given by Eqs (3) and (4), respectively:


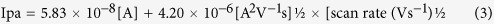



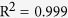



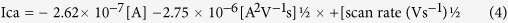



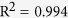


The anodic peak potential (E_pa_) varied linearly with the natural logarithm of the scan rate (ln ν), and were determined by the following equation:


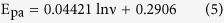


Laviron’s theory was used to estimate the surface concentration of the Ab/RGO/CA/Au electrode (Eq. 6), and the slope is given by Eq. (7):


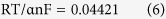



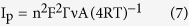


where α is the transfer coefficient, n is the number of electrons transferred (1 in this case), F is the Faraday constant (96485.34 Cmol^−1^), г is the surface concentration of the Ab/RGO/CA/Au electrode, ν is the scan rate (mV s^−1^), R is the gas constant (8.314J mol^−1^ K^−1^), and T is the absolute temperature (298 K). The I_p_/ν value can be calculated from the slope of the I_p_ versus ν plot. The total surface concentration of the Ab/RGO/CA/Au electrode was found to be 2.399 × 10^−9^ mol cm^−2^, demonstrating successful immobilization of antibody on the RGO/CA/Au surface.

The interfacial changes induced by the biological-recognition events were studied by a very sensitive tool, EIS. Figure 5(d) shows electrochemical impedance spectra of CA/Au (spectra i), RGO/CA/Au electrode (spectra ii), Ab/RGO/CA/Au electrode (spectra iii), and BSA/Ab/RGO/CA/Au electrode (spectra iv) for 10 mM PBS (pH 7.4) containing 2.5 mM [Fe(CN)_6_]^3−/4−^ and 100 mM NaCl, using 10 mV bias potential. The electrical impedance (Z) is the ratio of the incremental change in voltage, V(t), to the resulting change in current, I(t), and is given by Eq. (8).


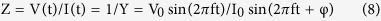


where V_0_ and I_0_ are the amplitudes of voltage and current signals, respectively, f is the frequency, t is time, φ is the phase shift between the voltage and current functions, and Y is the complex admittance. Impedance is described either by the modulus of Z and the phase shift φ or by its real (Z′) and imaginary components (Z″).

The charge transfer process in the electrodes was investigated by measuring the R_CT_ (charge transfer resistance) at the electrode/electrolyte interface. The R_CT_ value depends on the dielectric and insulating features at the electrode/electrolyte interface. The R_CT_ (3.2 kΩ) for CA/Au electrode (spectra i) decreased to 1.01 kΩ after RGO deposition (spectra ii). This was due to the conducting nature of the RGO compensating the insulating nature of CA. The R_CT_ value increased to 2.0 kΩ after antibody immobilization showing the insulating nature and macromolecular structure of antibody which hinders the electron transfer (spectra iii). A further increase in the R_CT_ value of 2.5 kΩ for BSA/Ab/RGO/CA/Au electrodes (spectra iv) indicates that BSA covered all the non-specific sites of Ab/RGO/CA/Au electrode and impeded the electron transfer.

Electrochemical response studies of the BSA/Ab/RGO/CA/Au electrode were carried out as a function of H1N1 virus concentration, using the chronoamperometry technique (Fig. 6(a)). During this measurement, the solutions containing various concentrations of H1N1 viruses in 10 mM PBS (pH 7.4) with 2.5 mM [Fe(CN)_6_]^3−/4−^ and 100 mM NaCl were injected through the inlet of the microchannel. It was observed that the chronoamperometric current increased proportionally with the H1N1 virus concentration in the range of 1 to 10^4^ PFU mL^−1^. A similar trend of increased current as a function of analyte concentration was observed in several studies of label-free electrochemical detection^35^,^36^,^37^. This may be due to the promotional spatial orientation of the antigen-antibody complexes, which might introduce effortless conducting ways for facile charge transfer to the electrode surface although no clear mechanism has been established yet^36^,^37^. Figure 6(b) shows the calibration plot between chronoamperometric current and virus concentration. The chronoamperometric current varied linearly with the injected virus concentration (from 1 to 10^4^ PFU mL^−1^), and the relationship was given by the following equation:


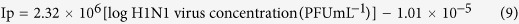



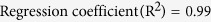


A control experiment was also conducted under the same conditions, using BSA/Ab/RGO/CA/Au electrodes without the virus (insets (ii) of Fig. 6(a,c), and Fig. S7). According to these measurements, this RGO-based chip showed an LOD of 0.5 PFU mL^−1^ based on a signal to noise ratio of 3, with a highly linear detection range of 1 to 10^4^ PFU mL^−1^. Taking into consideration that the concentration of influenza viral particles observed in the infected swine nasal samples is usually 10^3^–10^5^ TCID_50_ mL^−1^ (50% tissue culture infective dose)^38^, the present immunosensor is highly sensitive. In fact, Lee *et al*.^39^ reported an LOD of ~10^2^ TCID_50_ mL^−1^ using a carbon nanotube-based electrical biosensor. Zhang *et al*.^40^ also demonstrated an LOD of 10^2^ PFU for H1N1 viruses using disposable test strips within 1 h. The enhanced LOD in the present study can be due to the outstanding conducting property of RGO sheets which acts as an “electron wire” between the antibody and electrode surface, and the larger surface area of RGO sheets which provides oriented immobilization of antibody and facilitates redox reaction. The antibodies in the present study were immobilized in an oriented manner, not in a random manner, as there were plenty of carboxyl groups onto RGO sheets. That is, they were activated by EDC-NHS, and they were attached onto the sheets in upright condition, offering a well-oriented antibody onto the surface, which resulted in a high binding capacity as antigen-binding sites can be better accessible from the solution front in oriented-immobilization mode^41^. In fact, it was reported that less than 10% of antibodies remain active when immobilized in a random orientation as antibodies can be blocked due to incorrect antibody binding position^42^. The selectivity of the BSA/Ab/RGO/CA/Au electrode was also demonstrated using MS2 virus (10^4^ PFU mL^−1^) in the same buffer solution (Fig. 6c). In the test, the control electrode (BSA/Ab/RGO/CA/Au) showed a current almost similar to that of MS2/BSA/Ab/RGO/CA/Au. However, when the sensor was exposed to H1N1 viruses, the current was greatly increased. These measurements show that the present chip was very specific for influenza A H1N1 viruses, and imply that there was sufficient BSA on the sensor surface, as we used MS2 viruses having a concentration of the clinical detection range (10^4^ PFU ml^−1^).

## Conclusions

In this study, we developed a miniaturized microfluidics-integrated electrochemical immunosensor using RGO sheets for label-free detection of influenza H1N1 viruses, with immobilized antibodies via covalent bonding between NH_2_ end of antibody and carboxyl end of RGO which resulted into amide bond and improved the loading capacity of antibody onto the surface. TEM, RAMAN, XRD, and UV-VIS studies revealed successful RGO synthesis, and FTIR, SEM, AFM, and XPS studies confirmed the presence of functional groups required for antibody immobilization and virus capture. The lower intensities of the carboxyl group peak for Ab/RGO/CA/Au electrodes spectra and the emergence of amide peak implied that most of the carboxyl groups available on RGO were employed to form amide bond while immobilizing antibody. The microfluidics-integrated immunosensor showed a highly linear (R^2^ > 99%) behavior in the range of 1 to 10^4^ PFU mL^−1^ and improved LOD (0.5 PFU mL^−1^), along with high selectivity for H1N1 viruses, which was evidenced by the control experiments using MS2 bacteriophages. This RGO-based microfluidics-integrated electrochemical immunosensor can be employed to fabricate handheld multianalyte sensing devices for clinical diagnosis.

## Methods

### PDMS microchannel fabrication

PDMS has become an attractive hydrophobic polymeric material for the channel fabrication to precisely deliver target analytes, and provides good sealing properties via conformal contact with glass substrate, high optical transparency, chemical-compatibility with organic solvents with negligible swelling^43^,^44^.

An SU-8 mold for a microfluidic channel (width: 200 μm; height: 100 μm) was made on a silicon wafer (6 inch; i-Nexus, Korea), using SU-8 50 photoresist (MicroChem Corp, MA). After treating the mold surface with trichloromethylsilane for 30 min in vapor phase to ensure easy release, a mixture of PDMS elastomer and curing agent at a volume ratio of 10:1 (Dow Corning Corp, MI) was poured onto the SU-8 mold, cured in a dry oven at 60 °C for 3 h, and cut into chips. Upon use, the microchannel was peeled off from the mold, and inlet and outlet holes of the microchannel were made with a punch. Tubing was set up manually using tips and tubes, followed by connecting a syringe pump to the inlet.

### Microelectrode fabrication

Typically, electrochemical measurements necessitate a three-electrode set-up: a working electrode (WE), where the relevant binding occurs; a reference electrode (RE), which acts as a stable potential reference; and a counter electrode (CE), which collects the current between the WE and itself. A glass wafer (6 inch, i-Nexus, Korea) was used as a substrate for these three electrodes. Gold (Au) was chosen as the material for both WE and CE owing to its high standard electrode potential of 1.52 V and high resistance to corrosion. More complex aqueous electrodes, including Ag/AgCl and saturated calomel electrode, yield better potential stability, but are very difficult to integrate within a microfabricated device. Owing to the stable electrode potential and compatible nature with the nanofabrication process, platinum (Pt) was selected as an RE.

First, an RE was constructed on a glass substrate using a lift-off process. After a photoresist pattern for the RE was made using lithography, the substrate was pre-treated with oxygen plasma. A 10-nm thick layer of titanium, an adhesive layer, was sputter-deposited onto the glass surface, and then a 100 nm thick layer of platinum was deposited using electron-beam evaporation. Similarly, WE and CE were fabricated on the same substrate. A 10-nm thick layer of chromium, an adhesive layer, and a 100-nm thick layer of Au was electron-beam evaporated onto the substrate consecutively and substrate was washed with acetone to remove the residual photoresist. The width of each electrode was 500 μm, and the gaps between the adjacent electrodes were 2 mm. The wafer was then diced into chips (30 mm × 15 mm). The chips were sonicated in acetone for 15 min, followed by an additional 15 min sonication in distilled dH_2_O. These chips were then dried with gaseous nitrogen, and either used immediately for the fabrication of device, or stored under vacuum in a desiccator when not in use.

### Synthesis of RGO matrix

Natural graphite powder was oxidized to GO, according to the modified Hummer’s method^45^. RGO was then synthesized, according to an existing method, with a slight modification^46^. In a typical procedure, GO (1 mg mL^−1^) was first dispersed in 150 mL of dH_2_O, followed by sonication for 30 min at 25 °C. 750 mg of Mg(NO_3_)_2_.6H_2_O was dissolved in 150 mL of GO solution, followed by further sonication for 30 min at 25 °C. The GO solution was then constantly stirred at 80 °C, while adding a few drops of hydrazine hydrate, forming the RGO solution. The solution was cooled to room temperature after rapid stirring for 6 hours. The resulting black RGO solution was centrifuged at 8000 rpm for 20 min and washed 5 times with ultrapure water. The solution was then dispersed in 150 mL of water and kept at room temperature while not in use.

### Deposition of RGO onto the working electrode

The chips were cleaned using a piranha solution H_2_SO_4_:H_2_O_2_ (7:3) for a few seconds, followed by washing with dH_2_O, acetone, ethanol, and drying with nitrogen gas. The entire chip was muxed by a tape, except the WE. The chip was soaked in 10 mM CA aqueous solution in darkness at room temperature for 17 h to form a self-assembled monolayer onto the electrode. The chip was washed thoroughly with dH_2_O, ethanol, and dried using gaseous N_2_. The RGO solution was then deposited onto the electrode via dip coating for 24 h at room temperature, followed by washing with dH_2_O, ethanol, and dried with nitrogen gas.

### Biological tailoring onto the RGO/CA/Au electrodes using antibodies

**Figure 1:**
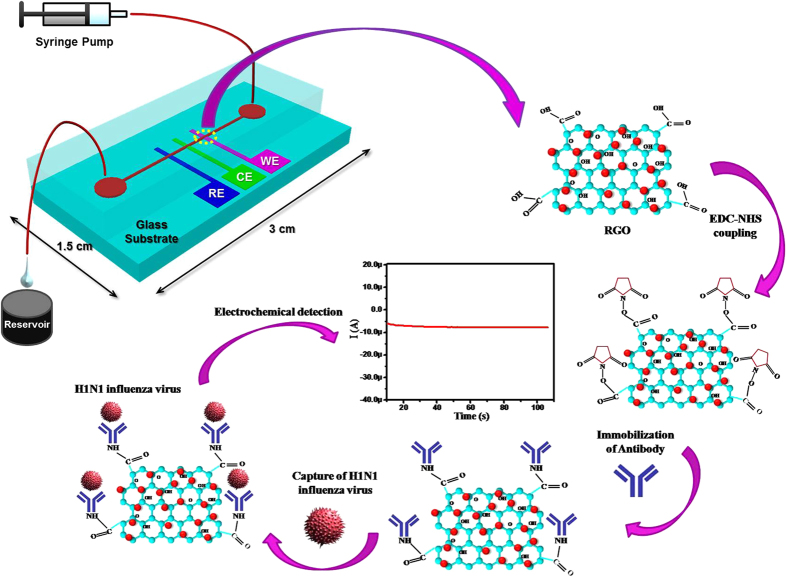
Schematic illustration of the microfluidics-integrated electrochemical immunosensing chip coated with RGO, followed by antibody immobilization using EDC/NHS coupling for the detection of influenza virus H1N1.

**Figure 2:**
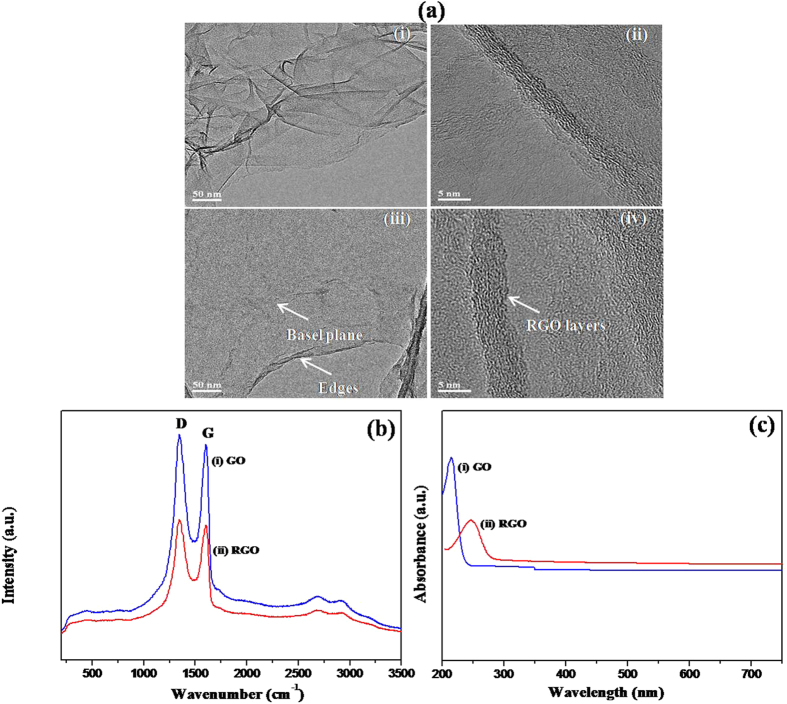
(**a**) Transmission electron micrographs of GO (i, ii) and RGO (iii, iv). A typical high-resolution transmission electron micrograph taken at the edges of RGO indicating several layers (~6–8). (**b**) Raman spectra of GO (i) and RGO (ii). (**c**) UV-visible spectra of GO (i) and RGO (ii).

**Figure 3:**
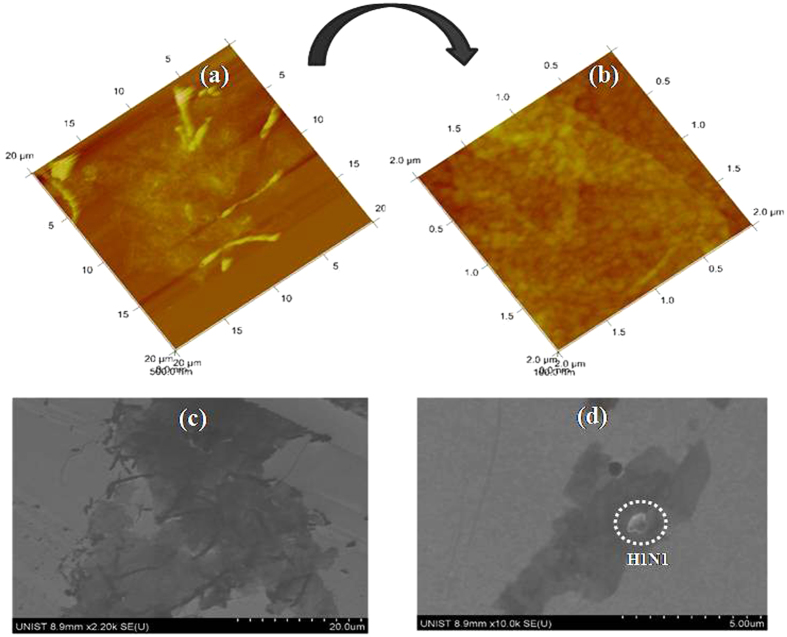
(**a**) Atomic force microscopic images of RGO/CA/Au. (**b**) Higher resolution of (**a**). (**c**) Scanning electron micrographs of RGO/CA/Au. (**d**) Ab/RGO/CA/Au after capture of H1N1 virus.

**Figure 4:**
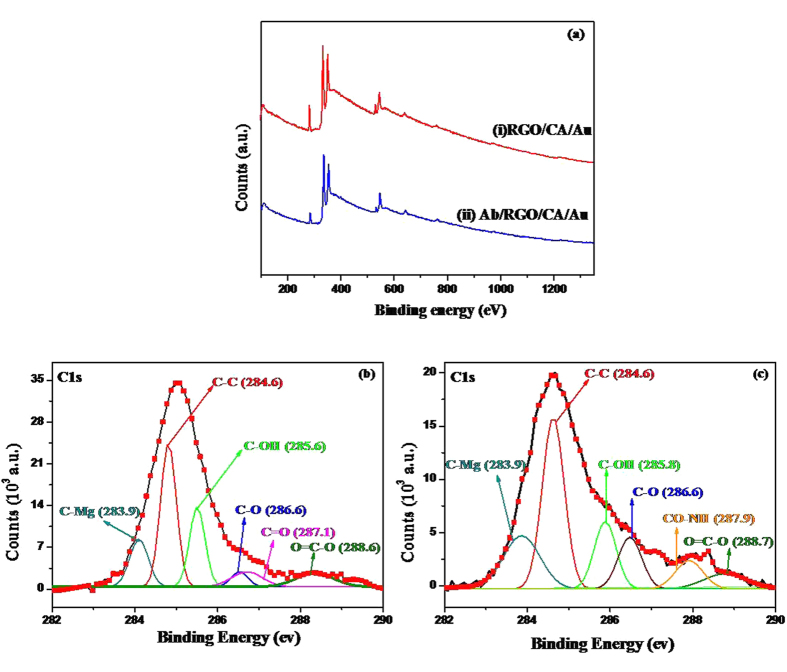
(**a**) Wide-scan X-ray photoelectron spectra of RGO/CA/Au (i) and RGO/CA/Au (ii). (**b**) X-ray photoelectron spectra of C1s region of the RGO/CA/Au electrode after deconvolution. (**c**) C1s region of the Ab/RGO/CA/Au electrode.

**Figure 5:**
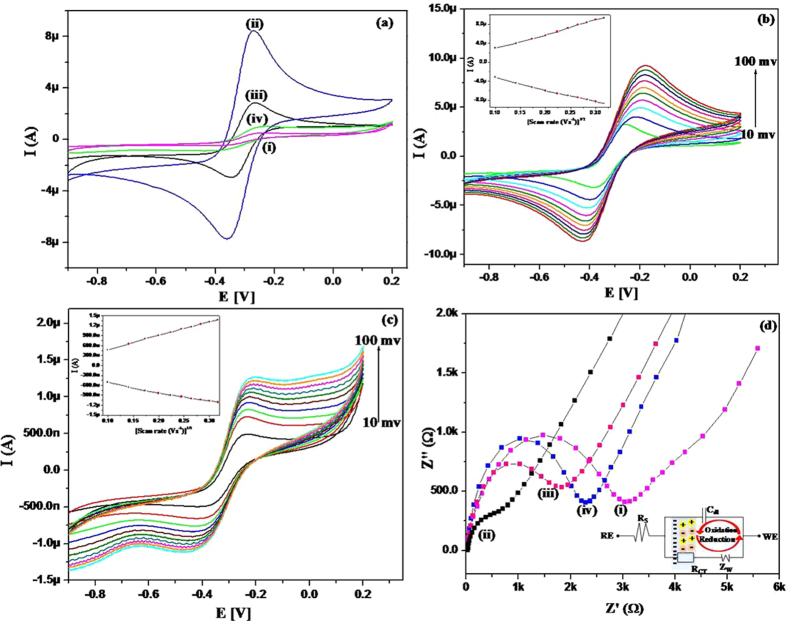
(**a**) Cyclic voltammogram of CA/Au (i), RGO/CA/Au (ii), Ab/RGO/CA/Au (iii), and BSA/Ab/RGO/CA/Au (iv) electrodes (**b**) Cyclic voltammogram of RGO/CA/Au electrode at different scan rates (10–100 mV s^−1^). (**c**) Cyclic voltammogram of BSA/Ab/RGO/CA/Au electrode at different scan rates (10–100 mV s^−1^) (inset: redox peak current as a function of the square root of the scan rate). (**d**) Electrochemical impedance spectra of CA/Au (i), RGO/CA/Au (ii), Ab/RGO/CA/Au (iii), and BSA/AB/RGO/CA/Au (iv) electrodes in 10 mM PBS (pH 7.4) containing 2.5 mM [Fe(CN)_6_]^3−/4−^ and 100 mM NaCl, (inset: a schematic representation of the Randles equivalent circuit model for impedance measurement).

**Figure 6:**
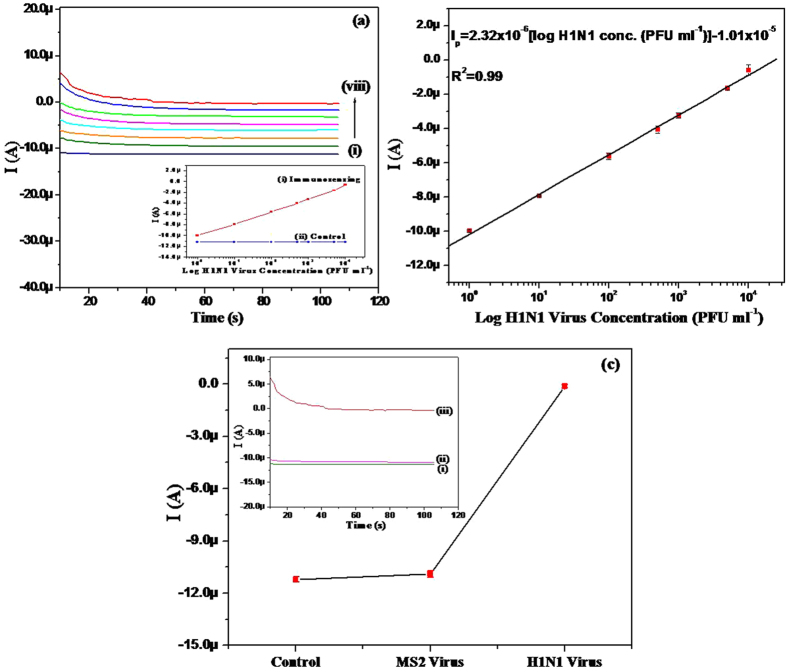
(**a**) Chronoamperometric response of the BSA/Ab/RGO/CA/Au-based immunochip as a function of H1N1 virus concentrations (1 to 10^4^ PFU mL^−1^) in a 10 mM PBS solution containing 2.5 mM [Fe(CN)_6_]^3−/4−^ and 100 mM NaCl. The experiment was controlled using a syringe pump attached to the inlet of the microsystem (inset: response current of BSA/Ab/RGO/CA/Au immunosensing chip with (i) or without (control) H1N1 virus concentration). (**b**) Calibration plot showing H1N1 virus concentrations (PFU mL^−1^) and the amperometric current of the immunochip during sensing. (**c**) Selectivity studies of the BSA/Ab/RGO/CA/Au immunochip against MS2 bacteriophages (10^4^ PFU mL^−1^) (inset: chronoamperometric graph of the control immunochip (i) incubated with MS2 bacteriophage (ii) or H1N1 virus (iii)).

The COOH group of the RGO/CA/Au electrode was activated by using EDC (5 μL, 15 mM) as a coupling agent and NHS (5 μL, 30 mM) as an activator (Fig. 1) for about 30 min at room temperature, followed by washing with PBS, water, and nitrogen gas drying. A solution of monoclonal antibody, specific to influenza virus H1N1 (10 μg mL^−1^), was freshly prepared in PBS (1x, pH 7.4). 10 μL of this solution was uniformly spread on the EDC/NHS activated surface of the RGO/CA/Au electrode, and was incubated at 37 °C for 2 h, followed by washing with PBS containing Tween 20, water, and drying with nitrogen gas. A strong amide (CO–NH) bond is expected to form between carboxyl groups of RGO and amino terminals of the antibody. BSA solution (1 mg mL^−1^) was used as a blocking agent for non-specific binding on the electrode. The chips were then washed with PBS and water, and used for virus capture.

### Microfluidic integration

The PDMS microchannels, prepared previously, were sealed on the glass chips using conformal contact (Fig. 1). The present chips consist of three electrodes: Ab/RGO/CA/Au electrode as the WE, Au/Cr electrode acting as the CE, and Pt/Ti electrode as the RE. The electrochemical studies of the chips were conducted using CV, EIS, and chronoamperometric techniques, and the virus concentration in 10 mM PBS (pH 7.4) containing 2.5 mM [Fe(CN)_6_]^3−/4−^ and 100 mM NaCl was varied from 1 to 10^4^ PFU mL^−1^, at a flow rate of 130 μL min^−1^.

### Characterizations

Structural characterization was performed using Fourier transform infrared (FTIR) spectroscopy (Varian 4100, UV-Vis-NIR (Liquid), Agilent, USA). Morphological studies were conducted using TEM (JEOL, USA), SEM (s-4800, Hitachi), and AFM (Dimension AFM 3100, Veeco, USA). For TEM studies, well suspended GO and RGO were prepared and drop-casted onto a carbon-coated copper holey grid. For SEM studies, the functionalized chips were coated with a thin layer of platinum to increase resolution, prior to SEM analysis. X-ray photoelectron spectrometer (Thermo Fisher, UK) equipped with an X-ray source and an α−110 hemispherical electron energy analyzer was used to conduct XPS measurements in the binding energy range of 0–1100 eV to confirm antibody immobilization. Raman spectra were recorded using Micro-Raman spectroscopy (WITec). High Resolution X-Ray Diffractometer (Bruker, Germany) was used to carry out X-ray diffraction (XRD) studies. An electrochemical analyzer (Autolab PGSTAT204, Metrohm, the Netherlands) was utilized to conduct electrochemical measurements in 10 mM PBS (pH 7.4) containing 2.5 mM of [Fe(CN)_6_]^3−/4−^ and 100 mM NaCl.

## Additional Information

**How to cite this article:** Singh, R. *et al*. Label-free Detection of Influenza Viruses using a Reduced Graphene Oxide-based Electrochemical Immunosensor Integrated with a Microfluidic Platform. *Sci. Rep.* **7**, 42771; doi: 10.1038/srep42771 (2017).

**Publisher's note:** Springer Nature remains neutral with regard to jurisdictional claims in published maps and institutional affiliations.

## Supplementary Material

Supplementary Information
