# Supplementary material for: Label-free Detection of Influenza Viruses using a Reduced Graphene Oxide-based Electrochemical Immunosensor Integrated with a Microfluidic Platform
Source: Sci Rep. 2017 Feb 15;7:42771. doi: 10.1038/srep42771 (PMC5309888; doi:10.1038/srep42771)
Supplement: Supplementary Information [file srep42771-s1.pdf]

# Supplementary Information

## Label-free Detection of Influenza Viruses using a Reduced Graphene Oxide-based Electrochemical Immunosensor Integrated with a Microfluidic Platform

Renu Singh[a], Seongkyeol Hong[a], Jaesung Jang[a,b,†]

[a]School of Mechanical and Nuclear Engineering, Ulsan National Institute of Science and Technology (UNIST), Ulsan 44919, Republic of Korea
[b]Department of Biomedical Engineering, UNIST, Ulsan 44919, Republic of Korea

[†] Correspondence should be addressed to jjang@unist.ac.kr. Tel: +82-52-217-2323 Fax: +82-52-217-2409

**1. X-ray diffraction (XRD) Analysis.** Fig. S1 shows an XRD pattern (copper Kα irradiation) of RGO in the range $10–35^{o}$. This pattern shows an intense and broad peak at $2\theta = 25.0^{o}$ that is assigned to (002) reflection plane, which confirms the formation of RGO [1].

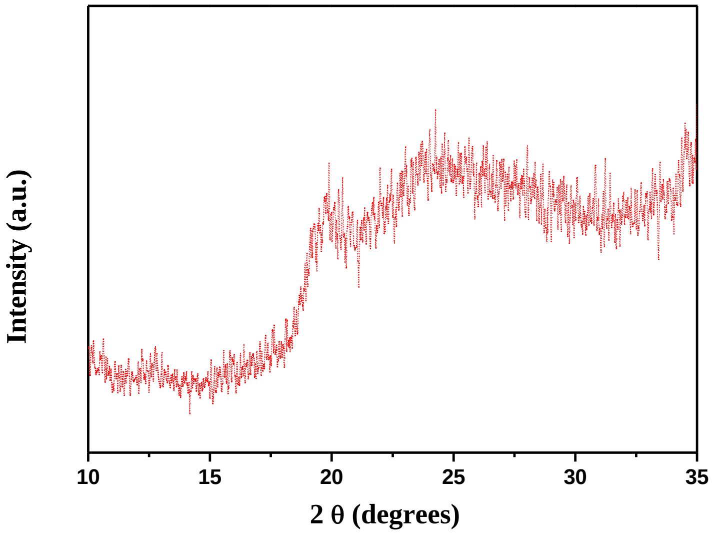


Fig. S1 X-ray diffraction (XRD) pattern of RGO

**2. FTIR spectroscopic analysis.** Fig. S2 shows FTIR spectra of RGO/CA/Au (spectra i) and Ab/RGO/CA/Au electrode (spectra ii). The bands seen at 1726 and 1410 $cm^{-1}$ are due to C=O stretching and O–H bending vibrations of the carboxyl group present in RGO. The band shown at 1645 $cm^{-1}$ is due to a C=C stretching mode, while the band observed at 1133 $cm^{-1}$ is due to C–OH stretching vibration [2]. C–H stretching and bending vibrations were observed at 2950 and 970 $cm^{-1}$, respectively. A broad band found at 3500 $cm^{-1}$ is due to O–H stretching vibration of the hydroxyl group present in the RGO [3]. (spectra i). After antibody immobilization onto the RGO sheet, the bands are shifted. Some extra bands seen at 1564 and 3505 $cm^{-1}$ are due to presence of amide A and amide II (overlapping with O–H stretch mode) in the antibody, indicating immobilization of the antibodies onto the RGO.

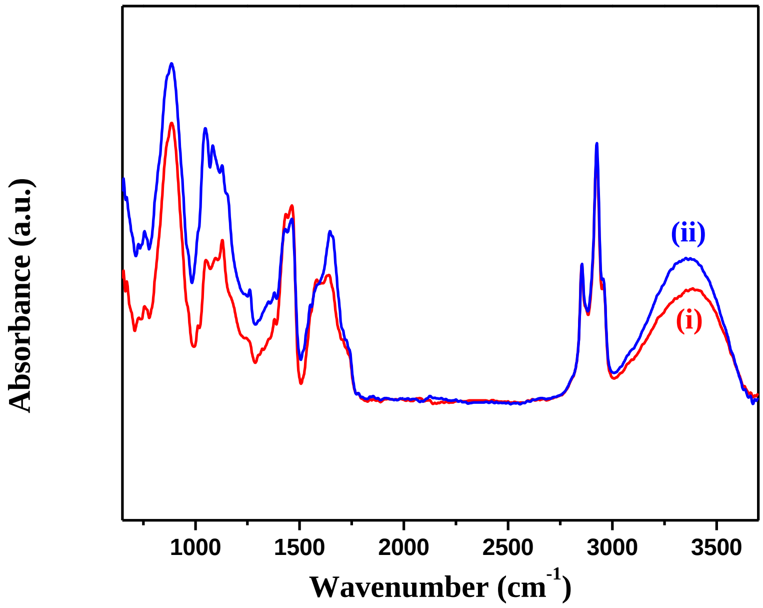


Fig. S2 Fourier transform infrared (FTIR) spectra of RGO/CA/Au (spectra i), and Ab/RGO/CA/Au (spectra ii) electrodes.

**3. RAMAN analysis**. Very sharp D peak noticed at 1350 $cm^{-1}$ corresponds to the bre athing mode of point phonons of A1g symmetry, which is attributed to the structural ( tearing and folding) disorder, point defects and existence of residual $O_2$ in the RGO. G-band, observed at 1590 $cm^{-1}$, shows E2g phonon of planar sp2-bonded carbon and o vertoned 2D-band spotted at 2685 $cm^{-1}$ corresponds to a second harmonic of D band which shows dispersive character as a function of excitation energy (Fig. 2(b)) [3].

**4. XPS analysis.**

**Table S1** Atomic concentrations (%) and binding energies (eV) of the elements C, N, O, and S present in the RGO/CA/Au and Ab/RGO/CA/Au electrodes, obtained from XPS analysis.

| Electrodes | Elemental quantification | | | | | | | |
|---|---|---|---|---|---|---|---|---|
| | C1s | | N1s | | O1s | | S2p | |
| | At (%) | BE (ev) | At (%) | BE (ev) | At (%) | BE (ev) | At (%) | BE (ev) |
| **RGO/CA/Au** | 53.92 | 285.3 | 0.23 | 400.8 | 7.54 | 532.6 | 4.00 | 162.7 |
| **Ab/RGO/CA/Au** | 39.22 | 285.3 | 6.41 | 440.2 | 6.21 | 532.3 | 5.29 | 162.5 |

Fig. S3 shows the XPS analysis for the N1s spectra of RGO/CA/Au and Ab/RGO/CA/ Au electrode. with the presence of a peak at 398.9 eV which attributes to the nitrog en atoms for Ab/RGO/CA/Au electrode and confirms efficient antibody immobilization, whereas the peak noted at 401.5 eV corresponds to the nitrogen approves covalent i mmobilization (Fig. S3(b,c)) [4].

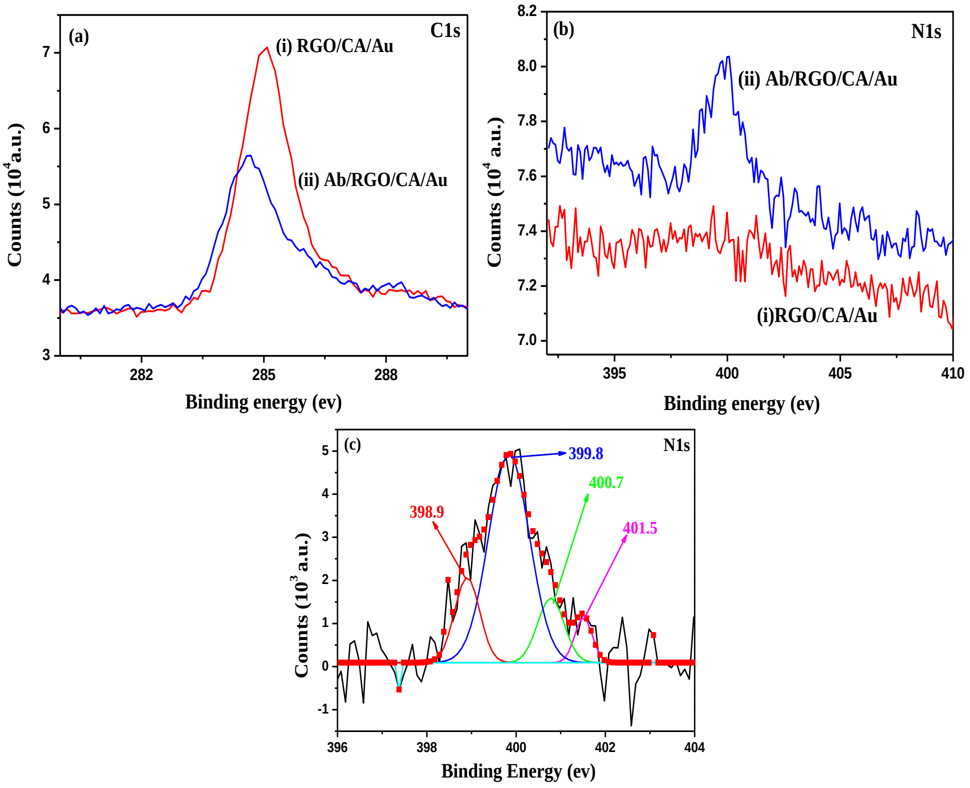


Fig. S3 XPS analysis of C1s (a) and N1 s (b) core level spectra of RGO/CA/Au (i) and Ab/RGO/CA/Au (ii). (c) N1s core-level spectra of the Ab/RGO/CA/Au electrodes.

**5. CV analysis.** Electrochemical CV studies of GO/CA/Au and RGO/CA/Au electrodes were conducted under 10 mM PBS (pH 7.4) containing 2.5 mM $[Fe(CN)_6]^{3-/4-}$ and 100 mM NaCl (Fig. S4). The RGO/CA/Au electrode exhibited well-behaved CV waves with sharp redox peaks, as compared to the rather broader redox peaks and lower response currents of GO. The magnitude of the anodic peak current for the GO/CA/Au electrode was 3.9 µA, and it increased to 8.4 µA for the RGO/CA/Au electrode. Moreover, the anodic peak potential of the GO/CA/Au electrode (-0.15 V) was found to be shifted towards a higher potential, as compared to that of the RGO/CA/Au electrode (-0.26 V), indicating the insulating nature of GO. Therefore, about two-fold increment in the peak current values, and a lower magnitude

of oxidation peak potential of RGO, revealed that RGO is a better candidate over GO for electrochemical sensing applications [5,6].

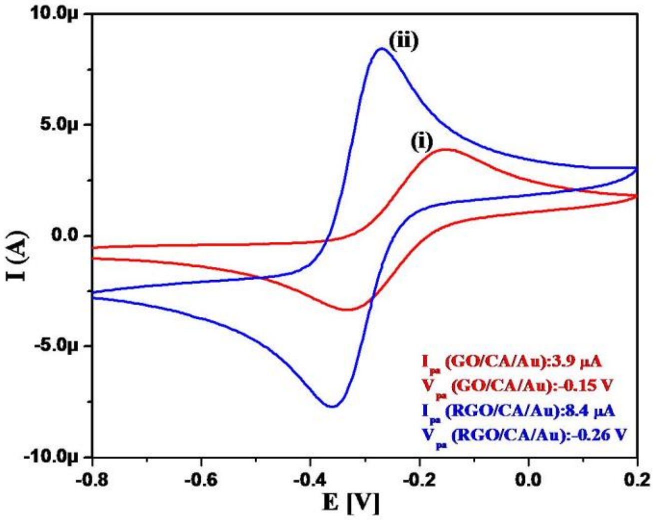


Fig. S4 cyclic voltammogram of GO/CA/Au (i) and RGO/CA/Au (ii) electrodes in PBS (pH 7.4) containing 2.5 mM $[Fe(CN)6]^{3-/4-}$.

**6. Optimization of antibody concentrations.**

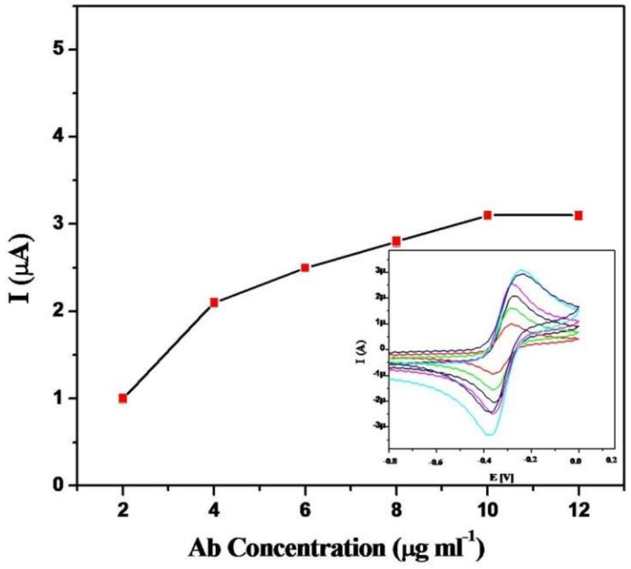


Fig. S5 Optimization curve for antibody concentration: response currents of Ab/RGO/CA/Au electrodes for various H1N1 specific antibody concentrations (inset: current vs. potential voltammograms for H1N1-specific antibody concentrations).

Antibody concentration was optimized by measuring the current responses as antibody concentrations of Ab/RGO/CA/Au electrodes were varied from 1 to 12 µg mL$^{-1}$ (Fig. S5). As the concentration of the antibodies immobilized onto the WE surface was varied from 1 to 10 µg mL$^{-1}$, the anodic peak current increased. This increase may be related to the fact that the antibodies strongly bound to the RGO modified Au surface through a covalent bond may promote the spatial orientation and affinity towards the antibodies. However, the current saturated at 10 µg mL$^{-1}$, as the active sites were filled by antibodies; hence, the surplus might have been discarded during washing. Therefore, 10 µg mL$^{-1}$ was used as the antibody concentration.

**7. Optimization of flow rate.** The flow rate of the media was also optimized for the BSA/Ab/RGO/CA/Au electrode under 10 mM PBS (pH 7.4) containing 2.5 mM $[Fe(CN)_6]^{3-/4-}$ and 100 mM NaCl using the chronoamperometric technique (Fig. S6). The chronoamperometric current response of the BSA/Ab/RGO/CA/Au electrode was obtained as a function of the flow rate (10–140 µL min$^{-1}$), and the corresponding current versus time is shown in the inset of Fig. S6.

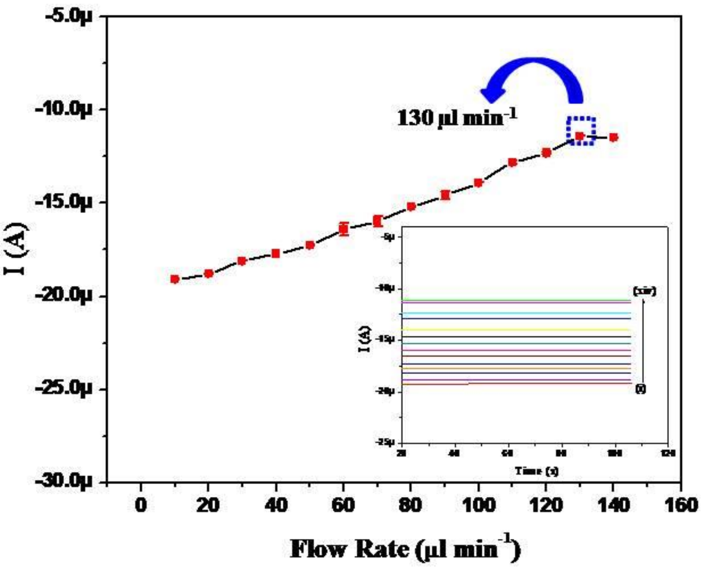


Fig. S6 Chronoamperometric response current versus flow rate (10–140 µL min$^{-1}$) plot of the BSA/Ab/RGO/CA/Au electrode (inset: chronoamperometric graphs of the flow rate).

It was observed that the chronoamperometric current increased with the flow rate. The maximum current was recorded at a flow rate of 130 $\mu L min^{-1}$. After this point, the amperometric current became fully saturated, and the response time of the electrode was also set to 300 s, as a safe choice (Fig. S6).

**8. Control experiment**

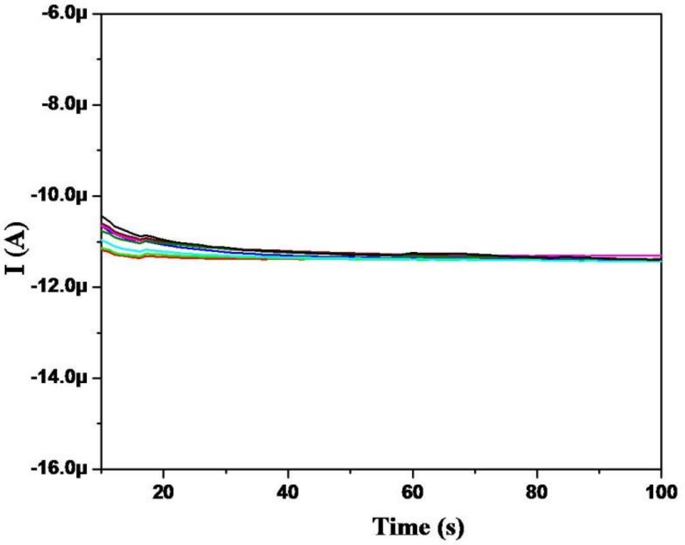


Fig. S7 Chronoamperometric response for control experiment of BSA/Ab/RGO/CA/Au electrode without any virus in PBS solution containing 2.5 mM $[Fe(CN)_6]^{3-/4-}$ and 100 mM NaCl.

**9. Electrochemical impedance spectroscopic analysis.** The most frequently used equi valent circuit for modeling the EIS experimental data is the Randles circuit (inset of Fi g. 5(d)), which consists of the electrolyte resistance ($R_S$) in series with an electric doub le-layer capacitance ($C_{dl}$), charge-transfer resistance ($R_{CT}$), and Warburg impedance ($Z_W$ ). The Nyquist plot shows a semicircular region lying on the real axis followed by a straight line. The linear portion ($\varphi = \pi/4$), observed in the low-frequency range, implie s a mass-transfer-limited process; the semicircular portion, observed in the high-freque ncy range, implies a charge-transfer-limited process. The imaginary component decreas es to zero at a high frequency because it offers no impedance. As the frequency drop s, the capacitance ($C_{dl}$) offers higher impedance and the current flows primarily throu

gh the $R_{CT}$ and $R_S$ segments.

**10. Chemicals and reagents.** Bovine serum albumin (BSA) (A2153), EDC (03449), NHS (130672), osmium tetroxide (75632), cystamine dihydrochloride (CA) (C8707), graphite flakes (332461), hydrazine solution (309400), trichloromethylsilane (92361), and isoamylacetate (112674) were obtained from Sigma–Aldrich (USA). Phosphate-buffered saline (PBS) (1x, pH 7.4) containing 0.1% Tween 20 (P2006), and magnesium nitrate (hexahydrate) ($Mg(NO_3)_2.6H_2O$) were purchased from Biosesang Inc. (South Korea). PBS (10x, pH 7.4, 70011-044) was also purchased from Invitrogen Life Technologies (USA). Acetone and hydrogen peroxide ($H_2O_2$) were procured from Samchun Pure Chemicals Co. Ltd. (South Korea). Sulfuric acid ($H_2SO_4$) was obtained from Junsei Chemical Co. Ltd. (Japan). Mouse anti-influenza A monoclonal antibody (OBT 1557) was purchased from AbD Serotec (USA). Influenza H1N1 viruses (KBPV-VR-33) were procured from the Bank of Pathogenic Viruses (South Korea). Bacteriophage MS2 (ATCC® 15597-B1™, $1 \times 10^9$ PFU $mL^{-1}$) was procured from Koram Biogen Corp. (South Korea). Deionized water ($dH_2O$) (resistance: ~18.2 MΩ) from the Millipore water purification system was used for preparation of the desired aqueous solutions (molecular biology grade). All solutions and glassware were autoclaved prior to being used.

## References


1 Park, S. *et al.* Colloidal suspensions of highly reduced graphene oxide in a wide variety of organic solvents. *Nano letters* **9**, 1593-1597, doi:10.1021/nl803798y (2009).

2 Paredes, J. I., Villar-Rodil, S., Martínez-Alonso, A. & Tascón, J. M. D. Graphene Oxide Dispersions in Organic Solvents. *Langmuir* **24**, 10560-10564, doi:10.1021/la801744a (2008).

3 Srivastava, R. K. *et al.* Functionalized multilayered graphene platform for urea sensor. *ACS nano* **6**, 168-175, doi:10.1021/nn203210s (2012).

4 Du, D. *et al.* Sensitive immunosensor for cancer biomarker based on dual signal amplification strategy of graphene sheets and multienzyme functionalized carbon nanospheres. *Anal Chem* **82**, 2989-2995, doi:10.1021/ac100036p (2010).

5 Wang, Z., Zhou, X., Zhang, J., Boey, F. & Zhang, H. Direct Electrochemical Reduction of Single-Layer Graphene Oxide and Subsequent Functionalization with Glucose Oxidase. *The Journal of Physical Chemistry C* **113**, 14071-14075, doi:10.1021/jp906348x (2009).

6 Pumera, M. Graphene-based nanomaterials and their electrochemistry. *Chemical Society Reviews* **39**, 4146-4157, doi:10.1039/C002690P (2010).
